# A study on intuitionistic fuzzy generating function using T-Norm, T-Conorm operators to enhance night-time images for autonomous driving system

**DOI:** 10.1038/s41598-025-15540-5

**Published:** 2025-09-29

**Authors:** M. S. Ragavendirane, S. Dhanasekar

**Affiliations:** Department of Mathematics, School of Advanced Sciences, Vellore Institute of Technology, Chennai, Tamilnadu 600127 India

**Keywords:** Autonomous driving systems, Intuitionistic fuzzy generator, Intuitionistic fuzzy set, T-Norm and T-Conorm operators, Mathematics and computing, Applied mathematics, Computational science, Computer science, Scientific data, Software

## Abstract

Enhancing night-time images is crucial for improving the performance of autonomous driving systems, which rely on high-quality visual input for accurate decision-making. This study explores the application of intuitionistic fuzzy generator in combination with T-Norm and T-Conorm operators to enhance low-visibility night-time images. Unlike traditional image processing methods, intuitionistic fuzzy set (IFS) incorporates both the degree of belonging and non-belonging aspects of an image allowing for a more detailed representation of uncertainty in image enhancement. The proposed method analyzes various aggregation operators, out of which Einstein’s T-Norm, Hamacher’s T-Conorm, Weber’s T-Conorm and W-probabilistic T-Conorm operators refine contrast, suppress noise and enhance illumination while preserving critical visual details. Extensive experiments on night-time driving datasets in contrast with existing state-of-the-art methods demonstrate that the recommended approach significantly improves image clarity via standard image quality metrics like SSIM, PSNR and correlation coefficient. Additionally, a sensitivity analysis conducted to assess the robustness and stability of the IFS components and aggregation operators with respect to the parameter $\Upsilon$ in validating its effectiveness in diverse low-light conditions. The findings indicate that integrating IFS with some particular T-Norm and T-Conorm operations is an innovative strategy to improve the autonomous vehicle’s perception in low-light conditions.

## Introduction

Recently, researchers have developed ADSs as an emerging technology that diversifies into various applications, such as object detection, tracking, segmentation and identification. An ADS involves the use of various sensors, algorithms and computer technologies to navigate and operate vehicles independently, without the need for human guidance. To develop an ADS, various components, such as cameras, light and radio detection with ranging, ultrasonic sensors, global positioning systems and an odometer are involved in the perceptional phase of the system. The system uses these components to identify and categorize obstacles, road conditions, pedestrians and traffic situations. ADSs are considered innovative results with societal and environmental benefits such as improved fuel economy, reduced traffic congestion and enhanced road safety^1^. The rapid growth of AI, deep learning and cross-sensor processing drives ADS’s development^2^.

Images play a crucial role in the ADS for object recognition and segmentation, as they provide visual data that is essential for the system in making accurate decisions and predictions to reduce the traffic accidents caused by human errors^3^. Furthermore, the integration of advanced image processing algorithms allows the ADS to not only detect obstacles and road conditions but also to differentiate between different types of objects such as vehicles, cyclists and pedestrians. They highly rely on cameras and sensors to perceive these environmental details. LLIE is used to efficiently improve the visual data by significantly improving the brightness, contrast and overall quality of images in dimly lit environments for ADSs. Recently, researchers have been working on making ADS better at seeing things, whereas ^4^ described a low-light image enhancement (LLIE) method for an ADS that uses deep learning and a multi-scale Retinex-based framework that uses Drive-Retinex-net. Deep neural networks, along with Retinex theory, yield optimized contrast enhancement and noise suppression in their illumination and reflectance components, which significantly improves night-time visibility for ADS. Moreover, the authors in^5^ designed a task-driven image enhancement network to improve high-level image restoration quality and performance. A network with little memory and a feature identity extraction module made computations simpler while keeping important features for perceptual tasks. Certainly, the authors in^6^ proposed a LE-net to enhance night-time driving images. LE-net integrates data augmentation, deep learning and attention mechanisms to enhance night-time images efficiently. In^7^, the authors provided a modified Bright Channel Prior and adaptive gamma correction to enhance visibility with minimal computational cost. It produces the most appropriate, natural-looking images effectively with low processing time and computational cost. Moreover,^8^ presents an end-to-end Retinex-based illumination that uses a multi-branch architecture and a memory gate mechanism to extract and preserve features at different depth levels. Then, in^9^, the authors suggested a framework based on convolutional neural networks. It has two parts: one that uses infrared images captured for ADSs to extract features and the other that improves the images. In^10^, the authors introduced a N-LoLiGan to enhance low-light tunnel images by removing contrast distortion. N-LoLiGan utilizes N-Net which integrates, a generator with a multi-scale input layer and a convolutional block attention module to improve illumination and color retention. Meanwhile,^11^ designed a real-time lane detection model for ADS during low-light conditions. In this model, zero-reference deep curve estimation++ is used as a LLIE tool to improve low contrast and correct exposure dynamically by predicting a set of luminance entropy curves that iteratively adjust pixel intensities for natural enhancement. Then, the authors in^12^ introduced a field programmable gate array based LLIE by incorporating Retinex algorithm and coarse grained re-configurable architecture which posses a low latency rate and power efficient solution beneficiary for various real time tasks. Recently,^13^ presented a real-time LLIE model to improve image visibility during the night which utilizes multi-stage Retinex-based decomposition for illumination enhancement and vision transformer for enhanced feature extraction. Further, adaptive light source-aware enlightenment and illumination-aware exposure-balanced fusion modules were employed to prevent over-enhancement by balancing overall brightness and contrast of the real night-time images. Thereafter, in^14^ the authors developed a fine grained vehicle type detector in an ADS using EfficientNet for low-light conditions where CLAHE and gamma corrections are utilized to improve the recognition capability. Recently,^15^ discussed about four major AI-based enhancement techniques: de-blurring, LLIE, de-raining, and dehazing to adverse some significant weather conditions like rain, fog, haze, darkness and motion blur which impacts the performance of ADSs.

Zadeh^16^ introduced fuzzy logic in 1965, which is excellent at dealing with uncertainty and lack of precision in numerous real-world cases. In image processing, it plays a predominant role while dealing with the vagueness and uncertainty in boundary pixels and spatial relations within the pixel intensities, which allows multiple levels of information representation from pixel-level classification to global scene understanding^17,18^.

In^19^, the authors introduced an automated image enhancement technique using a parametric index of fuzziness as an optimization criterion in finding the best S-function for contrast enhancement. Similarly,^20^ proposed a fuzzy based color image enhancement technique using Gaussian membership function and global contrast intensification operator which preserves original color composition and provides a balanced enhancement. Then,^21^ explored the potential applications of fuzzy logic in image enhancement and quality analysis due to its superior way of handling uncertainty in image processing inspired by the human way of reasoning. Meanwhile, in^22^ the authors proposed an automatic ridge-let image enhancement algorithm for road crack detection using fuzzy entropy and divergence which improves the contrast with reducing noise showing a better crack visibility. Certainly, the authors in^23^ discussed how effectively fuzzy cardinality can be measured and applied to image processing tasks such as color histograms and dominant color detection. The three fuzzy cardinality representations are explored and utilized for computational and linguistic descriptions which making them more robust and human-like.

However, the limitations of fuzzy set theory can be seen in certain cases where a higher level of precision and accuracy is required especially in ADSs to make decisions about navigation and obstacle avoidance based on varying degrees of certainty. Atanassov^24^ introduced the IFS in 1983 which addresses the uncertainty in a higher perspective, allowing for a more nuanced approach to handling imprecise information. By incorporating both membership and non-membership values, IFS can provide a more comprehensive representation of uncertainty and is successfully applied in various fields, including decision-making, pattern recognition and computer vision. Intuitionistic fuzzy image processing^25^ emerged to handle vagueness using membership, non-membership and hesitancy functions to better model uncertainty in image analysis. This approach has shown promising results in image segmentation, edge detection and image enhancement tasks such as, in^26^ proposed an IFS based segmentation approach incorporated with an additional hesitation factor to represent uncertainty in threshold selection and pixel classification.

Meanwhile,^27^ proposed an image fusion technique using IFS to improve the clarity, contrast and luminance of fused images. It utilizes IFS based entropy as an optimizing parameter for image fusion. The authors in^28^ introduced an image enhancement technique based on IFS in which hyperbolization is applied to amplify contrast differences between intensity levels. This kind of enhancement improves the visibility of images with weak edges by highlighting regions of interest while preserving image details. Similarly,^29,30^ proposed a new IFS based enhancement and clustering algorithm by utilizing a novel IFG to enhance low contrast mammogram images and to segment their lesions and tumors. Therefore, IFS-based techniques play a crucial role in improving the quality of medical images for better diagnosis and analysis. Based on these approaches,^31–34^ proposed a new way of enhancing low light and contrast images and image fusion by using Yager’s generating function^35^ and Chaira’s generating function^29^ which is integrated with traditional histogram equalization and contrast limited adaptive histogram equalization techniques. In a similar way,^36,37^ proposed a LLIE for images and videos using a new NIFG. Recently,^38^ introduced a LLIE model by using IFG and Retinex theory together resulting in a superior quality of images under low-light and extremely dark environments.

Aggregation operators are essential mathematical tools used to combine multiple input values into a single representative output, particularly in the presence of uncertain, imprecise and contradictory information. In the context of FS and decision-making, aggregation operations like T-Norms and T-Conorms are important because they help combine evaluations from different criteria and options into one overall score. In recent years, various types of aggregation operators have been introduced, such as those in^39,40^, which establish Aczel–Alsina power aggregation operators in the q-rung Orthopair fuzzy environment for decision-making problems involving complex-valued data, including dam construction site selection and stock market analysis. Similarly,^41,42^ established power aggregation and Dombi’s T-Norm and T-Conorm operators through the intuitionistic fuzzy rough sets for Dublin’s bike-sharing system and solar cell evaluation to handle uncertainty and imprecision better in the decision making environment. On the other hand, the authors in^43,44^ represented a novel multi-criteria decision-making framework for analyzing tuberculosis and Zika virus risk factors using Einstein and power aggregation operators in both arithmetic and geometric perspective for type-2 fuzzy sets and its extensions.

Due to their flexibility in integrating information and adaptability to various fuzzy environments, aggregation operators allow more robustness in the modeling of real-world complexities. Even in image processing,^45,46^ has introduced a medical image enhancement technique for CT scans, X-rays and pathological images in a type-2 fuzzy using the Hamacher’s T-Conorm aggregation operator. This operator combines the upper and lower membership functions of the type-2 fuzzy set, which show enhanced details and preserve important features of the image. Similar work done for MRI images in^47^ by using a modified type-2 fuzzy set with updating the value $\lambda$, which is the mean of the highest and lowest intensity of the input MRI image. In image enhancement, aggregation operators are used to integrate fuzzy information, which helps to improve quality of the image. It helps in combining contrast, brightness and edge information to produce an enhanced image.

**Fig. 1:**
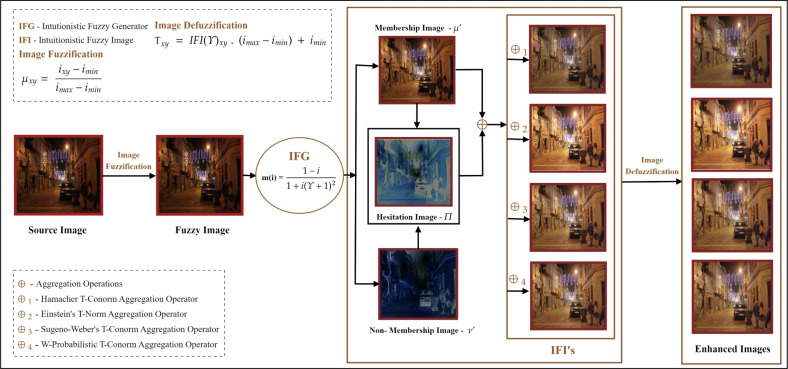
Overall framework of the proposed study.

As part of the literature review, different image enhancement algorithms were discussed based on various settings, including those based on machine learning, deep learning, fuzzy logic and IFS. These algorithms can be employed in ADS, medical diagnostics, surveillance and various other applications. This current study represents a new way of enhancing night-time road images for an ADS that integrates the intuitionistic fuzzy generating function with fuzzy aggregation operators such as T-Norms and T-Conorms to suppress noise and represent the uncertainty in pixel intensities far better than traditional enhancement techniques. Figure 1 shows the overall schematic representation of the recommended study. This paper contributes for LLIE of night-time images which has listed as follows:

- To introduce a new enhancement model for improving visibility in the night-time images for an ADS to detect obstacles using an intuitionistic fuzzy generator (IFG) associated with fuzzy T-Norm and T-Conorm operations.
- To demonstrate its superior performance, this study is compared with some traditional and contemporary enhancement algorithms such as, HE^48^, SSR^49^, MSR^50^, IFI^33^, IVIFI^31^, Chithra et. al.^36^, Ravindar et.al.^37^.
- To elaborate on its efficiency, the proposed method is validated through a comprehensive evaluation using standard image quality metrics like SSIM, PSNR and correlation coefficient.

The remaining paper is structured as follows: Table 1 comprises the abbreviations and acronyms utilized in this research, Section 2 presents the fundamental concepts required for this study, Section 3 outlines the proposed methodology of this research work, Section 4 includes the experimental analysis, and Section 5 provides the conclusion .

**Table 1:** List of abbreviations and acronyms.

S.no	Abbreviation / acronyms	Full Form
1.	ADSs	Autonomous driving systems
2.	CLAHE	Contrast-limited adaptive histogram equalization
3.	CT	Computed tomography
4.	HE	Histogram equalization
5.	FS	Fuzzy sets
6.	IFS	Intuitionistic fuzzy sets
7.	IFG	Intuitionistic fuzzy generator
8.	IFI	Intuitionistic fuzzy image
9.	IVIFI	Interval-valued intuitionistic fuzzy image
10.	LLIE	Low-light image enhancement
11.	LE-net	Light enhancement network
12.	LIDAR	Light detection and ranging
13.	MRI	Magenetic resonance imaging
14.	MSE	Mean squared error
15.	MSR	Multi-scale retinex
16.	N-LoLiGan	Novel low-light unsupervised generative adversarial network
17.	PSNR	Peak signal to noise ratio
18.	RADAR	Radio detection and ranging
19.	SOTA	State-of-the-art-techniques
20.	SSIM	Structural similarity index measure
21.	SSR	Single-scale retinex
22.	T-Norm	Triangular Norm
23.	T-Conorm	Triangular Conorm

## Fundamentals

### Fuzzy Set

Let $\mathbb {P}$ be any universe of discourse, a fuzzy set $\tilde{I}$ is a sub-collection of elements from $\mathbb {P}$ represented as $\tilde{I} = \{ (i,\mu _{\tilde{I}}(i)$)   $\forall \hspace{0.1cm} i \in \mathbb {P}\}$, where $\mu _{\tilde{I}}(i)$ denotes the membership function of *i* in $\tilde{I}$. The membership function $\mu _{\tilde{I}}(i)$ maps each element to a value in the interval [0, 1] which represents the degree of membership.

### Intuitionistic fuzzy set

Let $\mathbb {P}$ be the universe of discourse, an IFS $\tilde{I}_{int}$ on $\mathbb {P}$ defined as $\tilde{I}_{int} = \{(i,\mu _{\tilde{I}_{int}}(i), \nu _{\tilde{I}_{int}}(i)) \forall \hspace{0.16cm} i \in \mathbb {P} \}$, where $\mu _{\tilde{I}_{int}}(i)$ is called as the membership function and $\nu _{\tilde{I}_{int}}(i)$ is called as the non-membership function of i in $\tilde{I}_{int}$. Both $\mu _{\tilde{I}_{int}}(i)$ and $\nu _{\tilde{I}_{int}}(i)$ belongs to the closed interval [0,1] and $0 \le \mu _{\tilde{I}_{int}}(i) + \nu _{\tilde{I}_{int}}(i) \le 1$.

$\pi _{\tilde{I}_{int}}(i)$ is called the hesitation part or intuitionistic index and it is denoted by:

1 $$\begin{aligned} \pi _{\tilde{I}_{int}}(i) = 1 - (\mu _{\tilde{I}_{int}}(i) + \nu _{\tilde{I}_{int}}(i) ), \end{aligned}$$

where, $$0 \le \pi _{\tilde{I}_{int}}(i) \le 1$$.

Similarly, the membership and non-membership functions of an IFS denoted as,

2 $$\begin{aligned} \mu _{\tilde{I}_{int}}(i) = 1 - \nu _{\tilde{I}_{int}}(i) - \pi _{\tilde{I}_{int}}(i), \end{aligned}$$

3 $$\begin{aligned} \nu _{\tilde{I}_{int}}(i) = 1 - \mu _{\tilde{I}_{int}}(i) - \pi _{\tilde{I}_{int}}(i), \end{aligned}$$

where, $$\mu _{\tilde{I}_{int}}(i) + \nu _{\tilde{I}_{int}}(i) + \pi _{\tilde{I}_{int}}(i) = 1$$.

### Central tendency measures

Mean, median and mode represent the central tendency measures^51^ of an image, which summarize pixel intensity values, providing insights into its overall brightness and contrast by determining its statistical information.

Mean represents the arithmetic average of all pixel intensities within an image. It provides a measure of the overall brightness whereas higher mean signifies a brighter image and a lower mean indicates a darker one. Median is defined as the middle pixel intensity values from an image’s surrounding neighborhood after sorting them in ascending order. It gives a better representation of typical pixel intensity in a noisy image. Mode represents the pixel intensity that appears most frequently in an image which gives insight into the most dominant or recurring intensity value to identify common color, pattern and structure. The mean, median and mode of an input image *I* with $X \times Y$ dimension can be written as,

4 $$\begin{aligned} MEAN = \frac{1}{X \times Y}\sum _{x=1}^{X} \sum _{y=1}^{Y} i_{xy}, \end{aligned}$$

if $X \times Y$ is odd,

$$\begin{aligned} MEDIAN = \frac{X \times Y + 1}{2}, \end{aligned}$$

if $X \times Y$ is even,

$$\begin{aligned} MEDIAN = \frac{X \times Y}{2} + 1, \end{aligned}$$

5 $$\begin{aligned} MODE = arg max(I) \end{aligned}$$

### Triangular Norms and Conorms

T-Norms (*T*) and T-Conorm ($T^{*}$) are operations that extend the concepts of conjunction and disjunction to the realm of fuzzy logic^52^. T-Norms and T-Conorms are binary operators defined on the unit interval $[0,1]$, which are functions $[0,1]^{2} \rightarrow [0,1]$ that satisfy commutativity, associativity, monotonicity and possess an identity element.

- Let $\tilde{I_{1}},\tilde{I_{2}}$ be two fuzzy sets, then $T(i_{1},i_{2}) = T(i_{2},i_{1})$ ; $T^{*}(i_{1},i_{2}) = T^{*}(i_{2},i_{1})$.
- Let $\tilde{I_{1}},\tilde{I_{2}}$ and $\tilde{I_{3}}$ be three fuzzy sets, then $T(i_{1},T(i_{2},i_{3})) = T(T(i_{1},i_{2}),i_{3})$ ; $T^{*}(i_{1},T^{*}(i_{2},i_{3})) = T^{*}(T^{*}(i_{1},i_{2}),i_{3})$.
- If $$i_{2}<i_{3}$$, then $$T(i_{1},i_{2}) < T(i_{1},i_{3})$$ ; $$T^{*}(i_{1},i_{2}) < T^{*}(i_{1},i_{3})$$.
- $$T(i_{1},0) = i_{1}$$ ; $$T^{*}(i_{1},1) = i_{1}$$.

$$\forall$$ $$i_{1} \in \tilde{I_{1}}$$, $$i_{2} \in \tilde{I_{2}}$$, $$i_{3} \in \tilde{I_{3}}$$.

There are several T-Norm and T-Conorm operations in the literature proposed by Yager^53^, Dombi^54^, Weber^55^, Hamacher^56^ and Wang^57^.

Yager^53^ suggested the most demanding and the least interchangeable operations via T-Norm and T-Conorm as $Y(i_{1},i_{2})$ and $Y^{*}(i_{1},i_{2})$ follows.

6 $$\begin{aligned} Y(i_{1},i_{2})&= 1 - min([(1-i_{1})^{\Upsilon } + (1-i_{2})^{\Upsilon }]^{1/\Upsilon }, 1), \end{aligned}$$

7 $$\begin{aligned} Y^{*}(i_{1},i_{2})&= min([i_{1}^{\Upsilon } + i_{2}^{\Upsilon }]^{1/\Upsilon }, 1), \Upsilon > 0. \end{aligned}$$

Dombi^54^ has defined the a general class of fuzzy connectives on the basis of the connection between conjunctive and disjunctive to measure the fuzziness using T-Norm $D (i_{1},i_{2})$ and T-Conorm $D^{*}(i_{1},i_{2})$ as,

8 $$\begin{aligned} D(i_{1},i_{2})&= \dfrac{1}{1 + \left( \left( \frac{1}{i_{1}} - 1\right) ^{\Upsilon } + \left( \frac{1}{i_{2}} - 1\right) ^{\Upsilon }\right) ^{1/\Upsilon }}, \end{aligned}$$

9 $$\begin{aligned} D^{*}(i_{1},i_{2})&= \dfrac{1}{1 + \left( \left( \frac{1}{i_{1}} - 1\right) ^{-\Upsilon } + \left( \frac{1}{i_{2}} - 1\right) ^{-\Upsilon }\right) ^{-1/\Upsilon }}. \end{aligned}$$

Weber^55^ suggested a continuous T-Norm and T-Conorm fuzzy operation as follows

10 $$\begin{aligned} W(i_{1},i_{2})&= max(0,(1-\Upsilon )i_{1}.i_{2} + \Upsilon (i_{1} + i_{2} - 1)),\end{aligned}$$

11 $$\begin{aligned} W^{*}(i_{1},i_{2})&= 1 - W(1-i_{1} , 1-i_{2}). \end{aligned}$$

Hamacher^56^ developed a parameterized T-Norm $H(i_{1},i_{2})$ along with its dual T-Conorm $H^{*}(i_{1},i_{2})$ can be denoted as follows,

12 $$\begin{aligned} H(i_{1},i_{2})&= \dfrac{i_{1}.i_{2}}{\Upsilon + (1 - \Upsilon )(i_{1} + i_{2} - i_{1}.i_{2})},\end{aligned}$$

13 $$\begin{aligned} H^{*}(i_{1},i_{2})&= \dfrac{i_{1} + i_{2} - i_{1}.i_{2} - (1-\Upsilon )i_{1}.i_{2}}{1 - (1-\Upsilon )i_{1}.i_{2}}. \end{aligned}$$

To inspect any two membership function among IFSs, Wang^57^ introduced aggregation operators for Einstein T-Norms and T-Conorms given by $E(i_{1},i_{2})$ and $E^{*}(i_{1},i_{2})$,

14 $$\begin{aligned} E(i_{1},i_{2})&= \frac{i_{1} + i_{2}}{1 + i_{1}.i_{2}}, \end{aligned}$$

15 $$\begin{aligned} E^{*}(i_{1},i_{2})&= \frac{i_{1}.i_{2}}{1 - (1-i_{1})(1-i_{2})}. \end{aligned}$$

## Proposed methodology

**Fig. 2:**
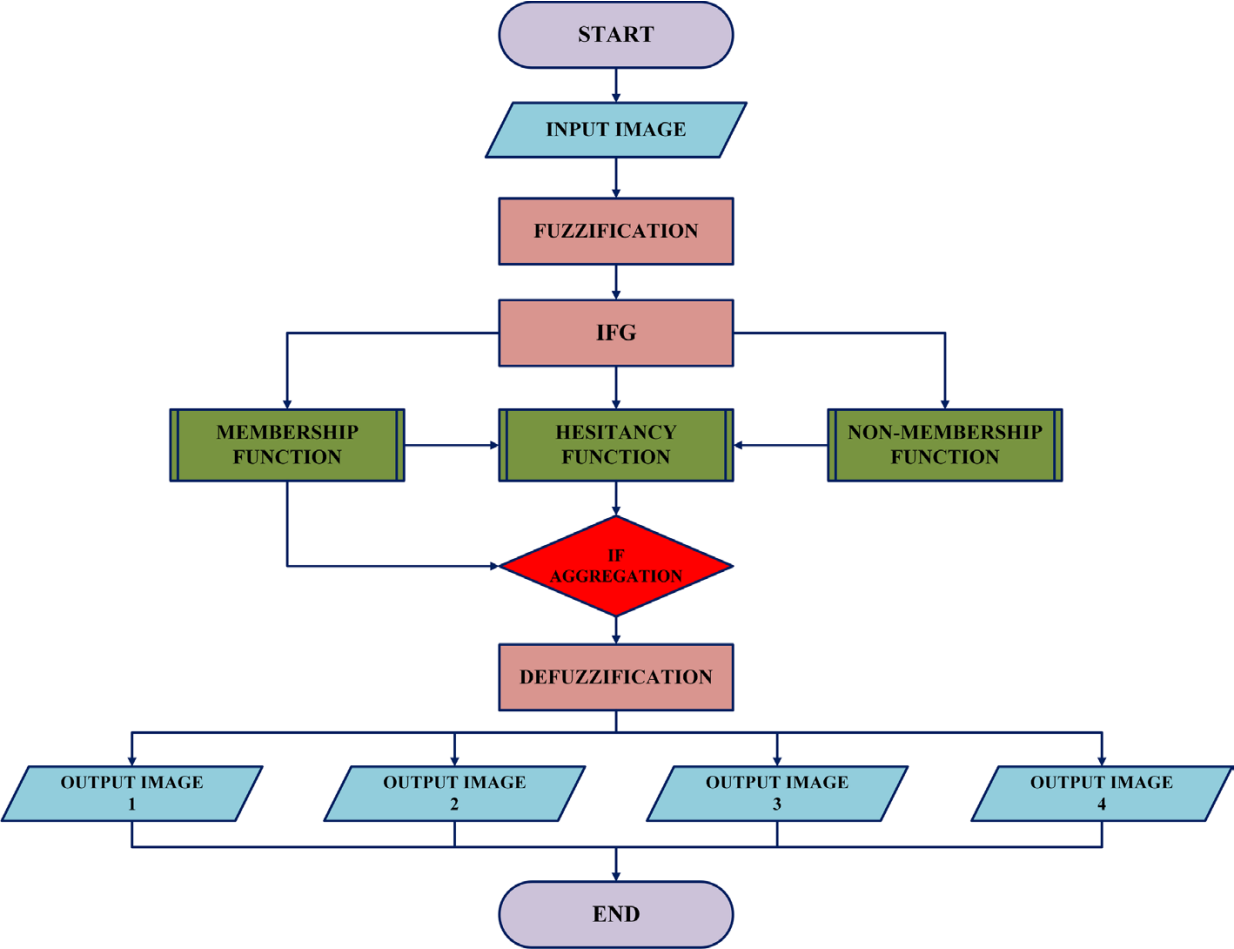
Flowchart of the proposed study.

A new way of operations introduced on images generated via intuitionistic fuzzy generating functions aggregated based on T-Norm and T-Conorm operators. This process involves (i) fuzzification, (ii) implementation of IFG’s followed by (iii) T-Norm and T-Conorm operations and (iv) estimation of $\Upsilon$ and finally (v) defuzzification to obtain the resultant enhanced images. The overall workflow of the proposed methodology is illustrated in Figure 2, which presents a structured flowchart depicting each step involved in the implementation process.

### Fuzzy image

The images are initially fuzzified using the normalization process which can also be determined as fuzzification whereas the domain of pixel intensities changes from [0,255] to [0,1].

The FI can be formulated in the $xy^{th}$ position as,

16 $$\begin{aligned} \mu _{xy} = \frac{i_{xy} - i_{min}}{i_{max} - i_{min} }, \end{aligned}$$

where $i_{xy}$ is the respective pixel level values of the original images, $i_{max}$ and $i_{min}$ denote the uppermost and lowermost pixel intensity values found in image *I*.

### Intuitionistic fuzzy generator^36^

On defining an IFG function for FS’s and IFSs. Any function $m:[0,1]\rightarrow [0,1]$   is an IFG

17 $$\begin{aligned} m(i)\le 1-i \hspace{0.2cm} \forall \,\,i \in [0,1],\nonumber \\ m(0)=1 \hspace{0.2cm} \& \hspace{0.2cm} m(1)=0. \end{aligned}$$

For every IFG $m:[0,1]\rightarrow [0,1]$, there exist a continuous function *h* such that $h(0)=0$ and their fuzzy complementary function can be written as,

18 $$\begin{aligned} m[\mu (i)] = h^{-1} [h(1) - h(\mu (i))]. \end{aligned}$$

19 $$\begin{aligned} h(\mu (i))&= \frac{1}{(\Upsilon +1)^{2}} log[1 + \mu (i)(\Upsilon + 1)^{2}] , \Upsilon > 0,\nonumber \\ h(0)&= \frac{1}{(\Upsilon +1)^{2}} log [1] = 0, \nonumber \\ h(1)&= \frac{1}{(\Upsilon +1)^{2}} log [1+(\Upsilon +1)^{2}], \end{aligned}$$

Considering $\mu (i)$ as *i* for simplicity,

20 $$\begin{aligned} h(i) = \frac{1}{(\Upsilon +1)^2} log[1 + i((\Upsilon +1)^2)]. \end{aligned}$$

The inverse function can be written as,

21 $$\begin{aligned} h^{-1} = \frac{e^{i(\Upsilon +1)^2} - 1}{(\Upsilon +1)^2} \end{aligned}$$

from (18), the inverse function (21) can be implemented as follows:

22 $$\begin{aligned} m(i) = h^{-1} \left( \frac{1}{(\Upsilon +1)^2} log \left( \frac{1+(\Upsilon +1)^2}{1+i(\Upsilon + 1)^2}\right) \right) , \end{aligned}$$

substitute (22) in (21) we get the IFG as,

23 $$\begin{aligned} m(i) = \frac{1-i}{1+i(\Upsilon + 1)^2} , \Upsilon > 0 . \end{aligned}$$

From (17), the membership function $\mu '_{xy}(i)$ can be written as,

24 $$\begin{aligned} \mu '_{xy}&= m(\mu _{xy}),\nonumber \\&= 1 - \frac{1-\mu _{xy}}{1 + \mu _{xy} (\Upsilon +1)^2}, \nonumber \\ \mu '_{xy}&= \frac{\mu _{xy}(1+(\Upsilon +1)^2)}{1 + \mu _{xy}(\Upsilon + 1)^2}. \end{aligned}$$

From (23), the non-membership function $\nu '_{xy}$ can be written as,

25 $$\begin{aligned} \nu '_{xy}&= m(\mu '_{xy}),\nonumber \\&= \frac{1 - \mu '_{xy}}{1+\mu '_{xy}(\Upsilon +1)^2}, \nonumber \\&= \dfrac{1 - \frac{\mu _{xy}(1+(\Upsilon +1)^2)}{1 + \mu _{xy}(\Upsilon + 1)^2}}{1 + \frac{\mu _{xy}(1+(\Upsilon +1)^2)}{1 + \mu _{xy}(\Upsilon + 1)^2} (\Upsilon + 1)^2},\nonumber \\ \nu '_{xy}&= \dfrac{1 - \mu _{xy}}{1 + \mu _{xy} (\Upsilon + 1)^2 (2 + (\Upsilon + 1)^2)}. \end{aligned}$$

Substitute (24) and (25) in (1), the hesitancy function $\Pi _{xy}$ can be written as follows,

26 $$\begin{aligned} \Pi _{xy}&= 1 - \mu '_{xy} - \nu '_{xy}, \end{aligned}$$

27 $$\begin{aligned}&= 1 - \frac{\mu _{xy}(1+(\Upsilon +1)^2)}{1 + \mu _{xy}(\Upsilon + 1)^2} - \dfrac{1 - \mu _{xy}}{1 + \mu _{xy} (\Upsilon + 1)^2 (2 + (\Upsilon + 1)^2)}, \nonumber \\&= \dfrac{1 + \mu _{xy} (\Upsilon + 1)^2 (2 + (\Upsilon + 1)^2) - (\mu _{xy}(1+(\Upsilon +1)^2)(2 + (\Upsilon + 1)^2)) - 1 + \mu _{xy} }{1 + \mu _{xy} (\Upsilon + 1)^2 (2 + (\Upsilon + 1)^2)}, \nonumber \\ \Pi _{xy}&= \dfrac{\mu _{xy}}{1 + \mu _{xy} (\Upsilon + 1)^2 (2 + (\Upsilon + 1)^2)}. \end{aligned}$$

### T-Norm and T-Conorm aggregation

**Fig. 3:**
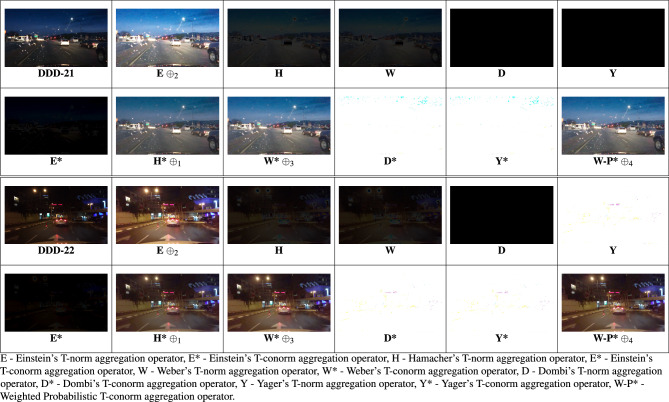
Images from BDD100k driving data set^58^ tested among various T-norm and T-conorm operators.

Section 2.4 discusses about different T-Norm and T-Conorm operators from the literature. To find the efficient operator for this analysis of the night-time driving images, they are evaluated by the some T-Norm and T-Conorm operators. Two images from^58^ are represented in the Figure 3, out of 10 existing operators, Hamacher’s T-Conorm $\oplus _{1}$, Einstein’s T-Norm $\oplus _{2}$, Weber’s T-Conorm $\oplus _{3}$ yields superior results in their enhancement.

From (13), the Hamacher’s T-Conorm aggregation $\oplus _{1}$ between $\mu '_{xy}$ and $\Pi _{xy}$ can be written as,

28 $$\begin{aligned} H^{*}(\mu '_{xy}, \Pi _{xy})&= \mu '_{xy} \oplus _{1} \Pi _{xy},\end{aligned}$$

29 $$\begin{aligned}&= \frac{\mu _{xy}(1+(\Upsilon +1)^2)}{1 + \mu _{xy}(\Upsilon + 1)^2} {\oplus _{1}} \dfrac{\mu _{xy}}{1 + \mu _{xy} (\Upsilon + 1)^2 (2 + (\Upsilon + 1)^2)}, \nonumber \\&= \dfrac{\mu '_{xy} + \Pi _{xy} + (\Upsilon -2)\mu '_{xy}.\Pi _{xy}}{1-(1-\Upsilon )\mu '_{xy}.\Pi _{xy}}. \end{aligned}$$

From (14), the Einstein’s T-Norm aggregation $\oplus _{2}$ between $\mu '_{xy}$ and $\Pi _{xy}$ can be written as,

30 $$\begin{aligned} E(\mu '_{xy}, \Pi _{xy})&= \mu '_{xy} \oplus _{2} \Pi _{xy},\end{aligned}$$

31 $$\begin{aligned}&= \frac{\mu _{xy}(1+(\Upsilon +1)^2)}{1 + \mu _{xy}(\Upsilon + 1)^2} {\oplus _{2}} \dfrac{\mu _{xy}}{1 + \mu _{xy} (\Upsilon + 1)^2 (2 + (\Upsilon + 1)^2)}, \nonumber \\&= \frac{\mu '_{xy}+\Pi _{xy}}{1 + (\mu '_{xy}.\Pi _{xy})}. \end{aligned}$$

From (11), the Weber’s T-Conorm aggregation $\oplus _{3}$ between $\mu '_{xy}$ and $\Pi _{xy}$ can be written as,

32 $$\begin{aligned} W^{*}(\mu '_{xy}, \Pi _{xy})&= 1 - W(1-\mu '_{xy}, 1-\Pi _{xy}) = \mu '_{xy} \oplus _{3} \Pi _{xy},\end{aligned}$$

33 $$\begin{aligned}&= \frac{\mu _{xy}(1+(\Upsilon +1)^2)}{1 + \mu _{xy}(\Upsilon + 1)^2} {\oplus _{3}} \dfrac{\mu _{xy}}{1 + \mu _{xy} (\Upsilon + 1)^2 (2 + (\Upsilon + 1)^2)}, \nonumber \\&= 1 - max(0, [(1-\Upsilon )(1-\mu '_{xy})(1-\Pi _{xy}) + \Upsilon ((1-\mu '_{xy})+(1-\Pi _{xy})-1)]). \end{aligned}$$

#### Weighted probabilistic T-Conorm operator

The W-Probabilistic T-Conorm aggregation $\oplus _{4}$ between $\mu '_{xy}$ and $\Pi _{xy}$ can be written as,

34 $$\begin{aligned} \mu '_{xy} \oplus _{4} \Pi _{xy}&= \frac{\mu _{xy}(1+(\Upsilon +1)^2)}{1 + \mu _{xy}(\Upsilon + 1)^2} {\oplus _{4}} \dfrac{\mu _{xy}}{1 + \mu _{xy} (\Upsilon + 1)^2 (2 + (\Upsilon + 1)^2)}, \nonumber \\&= \mu '_{xy} + \Pi _{xy} - (1-\Upsilon )^{2} .\mu '_{xy}.\Pi _{xy}. \end{aligned}$$

### Estimation of ’$\Upsilon$’

The corresponding image’s *mode* value, which is to be processed, is determined to be the parameter value $\Upsilon$ since the mode finds the dominant pixel that occurs in an image and determines its intensity. This current study considers $\Upsilon$ between [0, 1]. Therefore, the fuzzy image’s (16) *mode* is calculated and applied in the operations and generating functions. The proposed study initiates flexibility in choosing the $\Upsilon$ value for every image to be enhanced.

### Defuzzification

Defuzzification is the process of transforming a fuzzy set, which represents uncertain or imprecise information, into a desired output. In image processing, it involves converting fuzzy values representing degrees of membership for different elements within an image into precise values. Fuzzy logic systems often handle images by addressing uncertainty and ambiguity through a variety of operations. Defuzzification translates the resulting fuzzy values into a well-defined output, such as specific pixel intensities tailored to the image’s context, after applying these fuzzy operations.

From (16), each pixel in the image *T* defuzzified as,

35 $$\begin{aligned} T_{xy} = IFI(\Upsilon )_{xy}(i_{max} - i_{min}) + i_{min}. \end{aligned}$$

## Experimental analysis

The experiments were executed using MATLAB (R2023a) with the Image Processing Toolbox. The machine used for the setup is equipped with a Ryzen 5 7520U processor (AMD technology) and Radeon graphics, operating at 2.80 GHz. It runs on Microsoft Windows 11 Home and includes 512GB of SSD storage and 16GB of RAM.

**Fig. 4:**
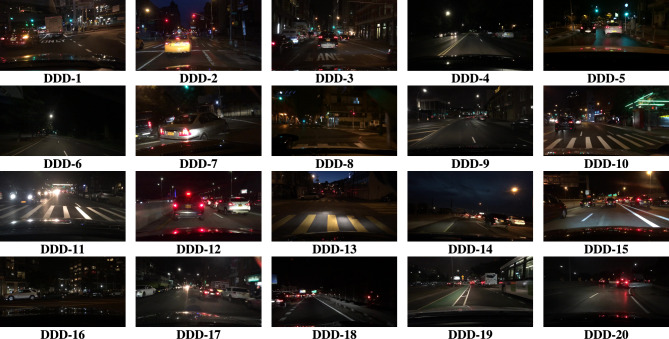
Source images from BDD100k driving data set.

**Fig. 5:**
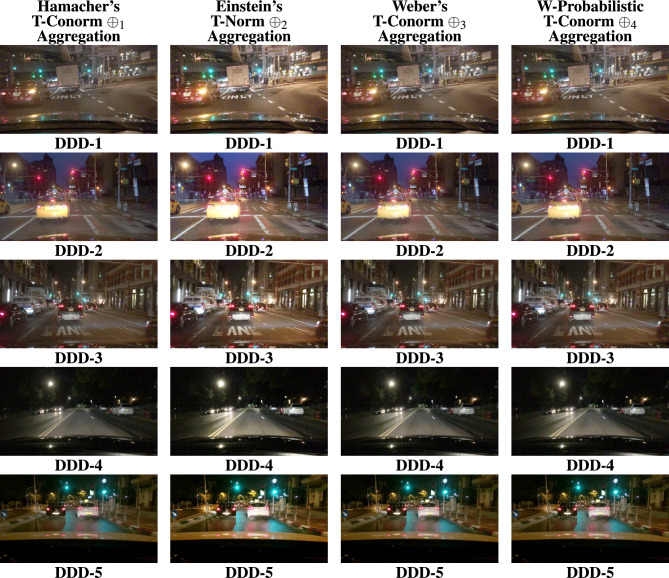
Enhanced images from BDD100k driving data set.

**Fig. 6:**
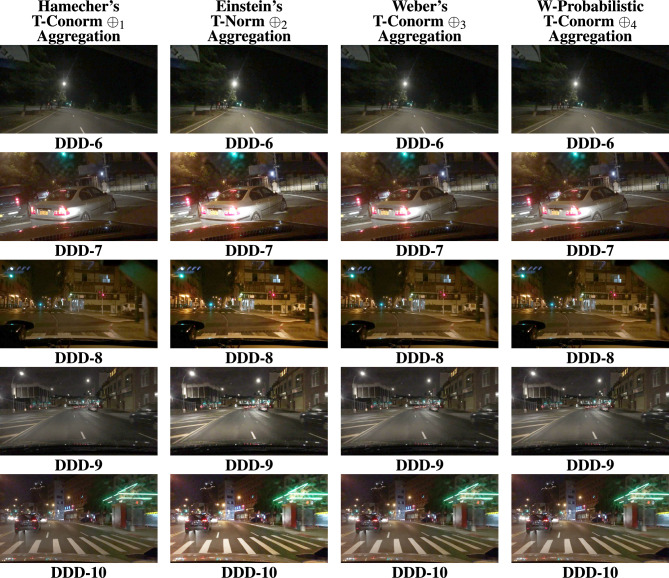
Enhanced images from BDD100k driving data set.

**Fig. 7:**
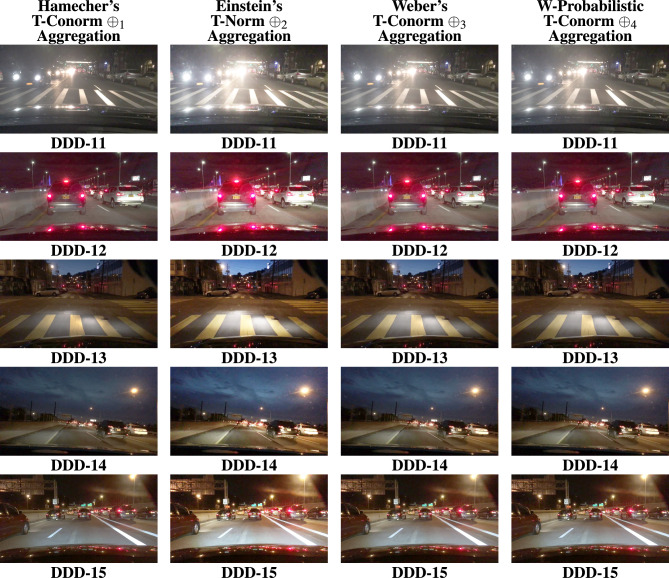
Enhanced images from BDD100k driving data set.

The BDD100K^58^ driving image data set is utilized for the entire analysis part of this work. Nearly thousands images were evaluated for this study to show how efficient the proposed study performs apart from other SOTA. 20 images were represented as DDD - 1, 2, ..., 20 in the Fig. 4. The results are primarily processed by performing the derived aggregation operations $\oplus _{1}$, $\oplus _{2}$, $\oplus _{3}$ and $\oplus _{4}$. Each operator yields enhanced image and the resultant 4 images generated by the suggested operators is compared with existing methods in the literature such as,^33,34,36,37,48–50^. Figures 5, 6, 7, & 8 represents the enhanced version of the images DDD −1, 2, ..., 20 by the Hamacher’s T-Conorm $\oplus _{1}$, Einstein’s T-Norm $\oplus _{2}$, Weber’s T-Conorm $\oplus _{3}$ and W-Probabilistic T-Conorm $\oplus _{4}$ aggregation operators. To evaluate the efficiency of the proposed study, the standard image quality metrics such as SSIM, PSNR and correlation coefficient are utilized here and compared with other existing methods and illustrated in the Table 2.

**Fig. 8:**
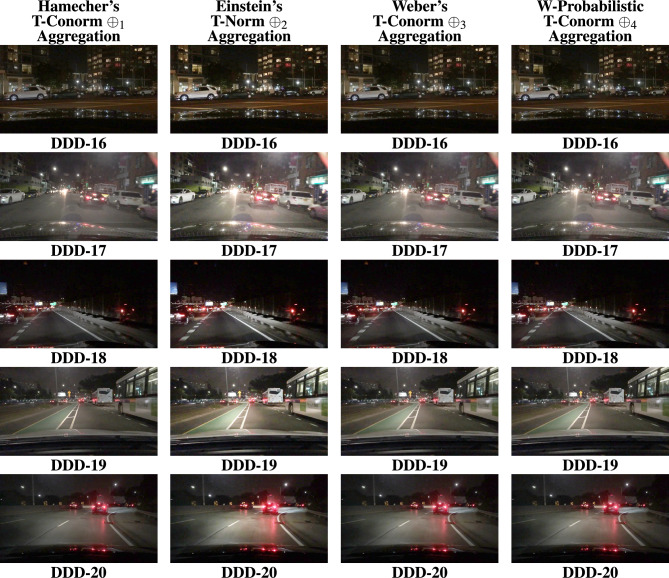
Enhanced images from BDD100k driving data set.

**Fig. 9:**
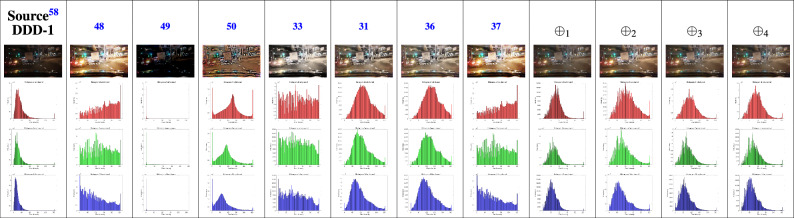
Histogram of image DDD-1 by proposed and state-of-the-art techniques.

A histogram is a graphical representation that shows frequency distributions of pixel intensities in an image. It provides a visual summary of how often each intensity value occurs, helping to analyze and understand the image’s characteristics. Figure 9 represents the histograms for the source image DDD-1 along with its comparison methods, such as HE^48^, SSR^49^, MSR^50^, IFI^33^, IVIFI^31^, Chithra et al.^36^, Ravindar et al.^37^ and the proposed $\oplus _{1}$, $\oplus _{2}$, $\oplus _{3}$, $\oplus _{4}$ operators generated images via red, green and blue color channels. From this figure, it is visually observed that the histogram pattern of the images generated by operators was similar and amplified compared to other state-of-the-art techniques. The resultant images also present a clear view of the environment, especially at night-time.

### Structural similarity index measure

The Structural Similarity Index Measure (SSIM) is a metric for assessing the similarity between two images. It’s specifically crafted to gauge the structural similarity, luminance and contrast details between an original image and a modified version. SSIM finds widespread application in image processing and computer vision, particularly for evaluating the effectiveness of techniques such as image and video compression, denoising and various image enhancement methods. An SSIM score close to 1 indicates high perceptual similarity, while a lower score indicates noticeable degradation.

36 $$\begin{aligned} SSIM(I,T) = \frac{(2{\zeta _{1}} {\zeta _{2}} + x_1)(2{\lambda _{12}} + x_2)}{({{\zeta _{1}}^2} + {{\zeta _{2}}^2}+ x_1)({\lambda _{1}}^{2} +{\lambda _{2}}^{2} + x_2)}, \end{aligned}$$

where $x_1$ and $x_2$ are non-negative constants.

$\zeta _{1}$ and $\zeta _{1}$ denotes the average gray levels of images *I* and *T*.

$\lambda _{1}$ and $\lambda _{2}$ denotes the variance of *I* and *T*.

$\lambda _{12}$ indicates the covariance of *I* and *T* respectively.

In Table 2, the average SSIM values are demonstrated for 1000 images and the proposed study is compared with existing methods such as,^33,34,36,37,48–50^. By the definition, the images generated by $\oplus _{1}$, $\oplus _{3}$, $\oplus _{4}$ yields average SSIM values of **0.5967**, **0.5432**, & **0.5414** which is maximum compare to all other methods. It shows that the structural information of the images generated by the proposed study are preserved compared to other existing methods. The average SSIM values are graphically represented in Figure 11a .

### Peak signal-to-noise ratio

PSNR is a metric used to assess the quality of a reconstructed image by comparing it to the original. Measured in decibels (dB), PSNR is derived from the mean squared error (MSE) between corresponding pixels of the two images. A higher PSNR value indicates closer similarity to the original image, reflecting lower distortion and higher fidelity. This metric is usually applied in image processing and compression to evaluate the performance of algorithms and techniques.

37 $$\begin{aligned} PSNR = 10.log_{10}\frac{{max(T)}^2}{MSE} , \end{aligned}$$

where, MSE stands for mean squared error and implies,

$$\begin{aligned} MSE = \frac{1}{X \times Y}\sum _{x=0}^{i-1} \sum _{y=0}^{j-1} (I_{xy} - T_{xy})^{2}. \end{aligned}$$

**Fig. 10:**
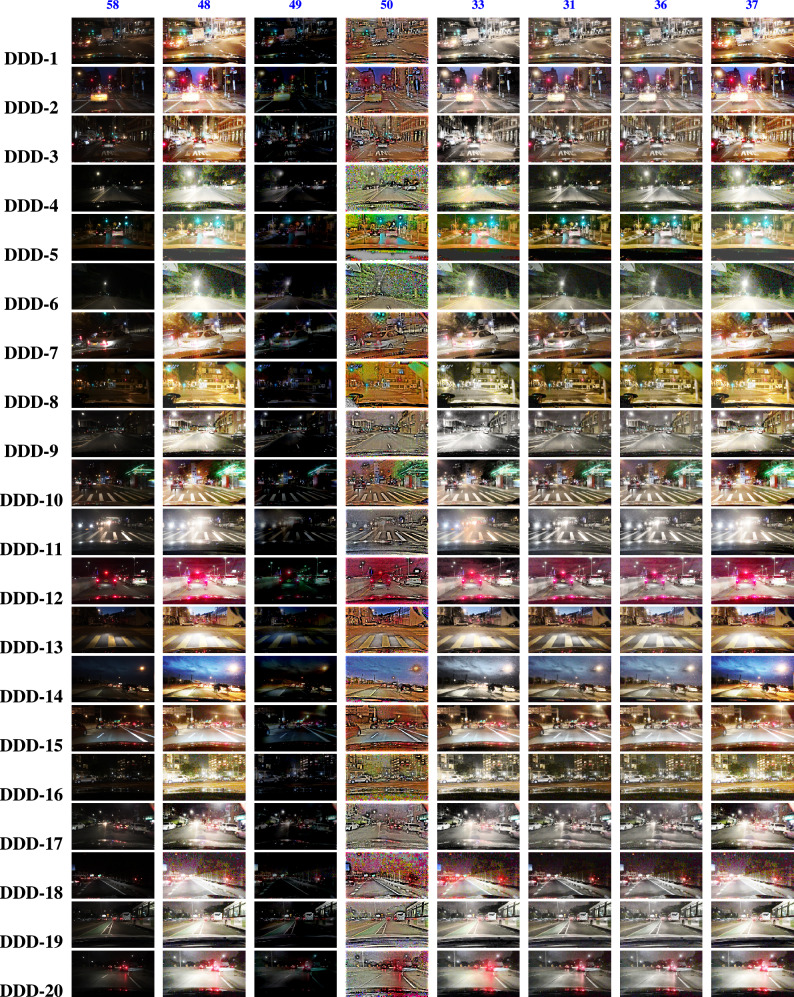
Some BD100K images via SOTA methods.

**Fig. 11:**
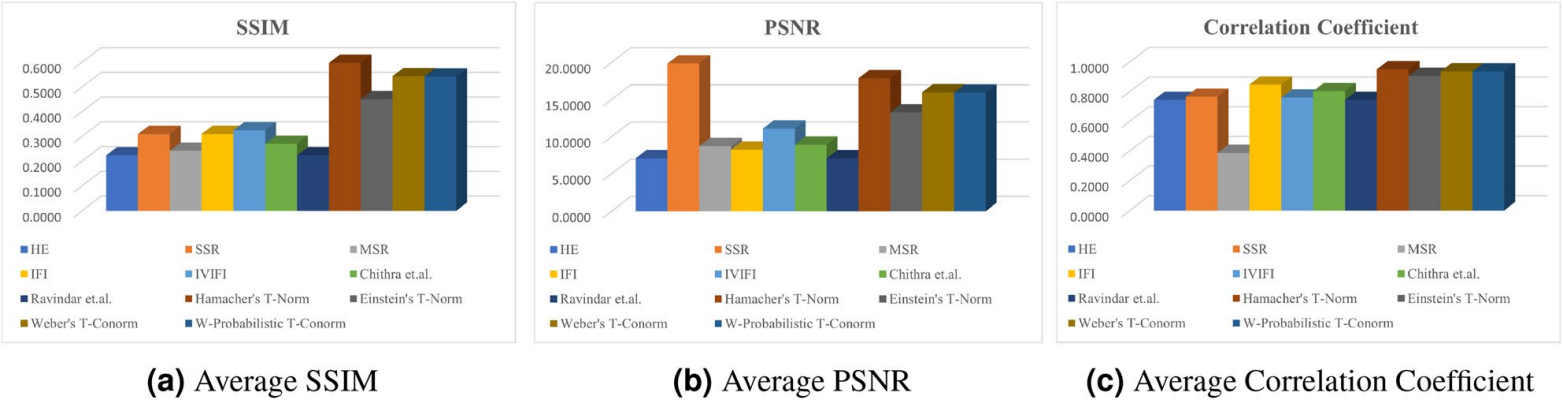
Graphical representation of SSIM, PSNR, Correlation Coefficient.

Table 2 represents the average PSNR value of the images from BDD100k^58^ dataset compared with existing state-of-art-techniques and the proposed study. $\oplus _{1}$ and $\oplus _{3}$ yields the second and third highest PSNR score of **17.8326** & **15.9449** where SSR^49^ yields the maximum value which is **19.8173**. It is visually observed in the Figure 10 that images generated by SSR is too dark compared to the source image itself hence PSNR is showing high values compared to the proposed study. Figure 11bshows the graphical illustration of average PSNR values.

### Correlation coefficient

In the field of imaging, the correlation coefficient is a statistical metric employed for evaluating the similarity between two images based on their pixel intensity levels. It gauges the intensity and direction of a linear relationship between two images. It quantifies the degree to which one image’s pixel values correspond to those of another image.

38 $$\begin{aligned} r = \frac{\sum (I_{xy} - \text {mean}(I)) \cdot (T_{xy}- \text {mean}(T))}{\sqrt{\sum (I_{xy} - \text {mean}(I))^2} \cdot \sqrt{\sum (T(x,y) - \text {mean}(T))^2}}. \end{aligned}$$

Unlike SSIM, correlation coefficient justifies how closely the pixel values of the enhanced image align with those of the source image. In Table 2, the average correlation coefficient of existing methodologies compared with the proposed study. It shows that the images generated by $\oplus _{1}$, $\oplus _{2}$, $\oplus _{3}$ and $\oplus _{4}$ yield maximum average correlation coefficient value greater than **0.9** which denotes that the enhanced images retains the pixel intensity relationships of the source image, suggesting high-quality enhancement. The correlation coefficient values are well illustrated in the Figure 11c.

**Table 2:** Comparison of SSIM, PSNR and Correlation with proposed and SOTA methods.

Methods	SSIM $\uparrow$	PSNR $\uparrow$	Correlation $\uparrow$
^48^	0.2228	7.0693	0.7430
^49^	0.3088	*19.8173*	0.7641
^50^	0.2416	8.7158	0.3856
^33^	0.3090	8.2332	0.8461
^31^	0.3244	11.0732	0.7583
^36^	0.2699	8.8917	0.8020
^37^	0.2228	7.0693	0.7429
$\oplus _{1}$	*0.5967*	***17.8326***	*0.9492*
$\oplus _{2}$	0.4497	13.2439	0.9049
$\oplus _{3}$	***0.5432***	15.9449	***0.9347***
$\oplus _{4}$	0.5414	15.8949	0.9341

’italic’ indicates the highest value.

’bolditalic’ indicates the second highest value.

’underline’ indicates the third highest value.

The texts in Bold indicates the proposed methodologies.

$\oplus _{1}$, $\oplus _{2}$, $\oplus _{3}$ and $\oplus _{4}$ are proposed approaches

The experimental analysis were executed for the images in^58^ and compared with some existing methodologies, such as^33,34,36,37,48–50^ by means of some standard image quality metrics like SSIM, PSNR and Correlation coefficient. The proposed methods yields a superior value and the images of $\oplus _{1}$, $\oplus _{2}$, $\oplus _{3}$ and $\oplus _{4}$ were demonstrated in the Figure 5, 6, 7 and 8. The graphical representation of the image quality metric values is shown in the Figure 11. Images generated by $\oplus _{1}$, $\oplus _{2}$, $\oplus _{3}$ and $\oplus _{4}$ posses superior quality, unlike traditional techniques, this kind of enhancement can be more efficiently utilized by an ADS to improve the visibility of any particular environment to detect obstacles.

### Sensitivity analysis

Sensitivity analysis^59^ is a technique that determines how variations in input parameters influence the output of a given model. In this enhancement model, $\Upsilon$ is utilized as the parameter which is associated with improving the visibility of night-time driving images^58^. By measuring the sensitivity index, one can assess which parameters exert the most influence on the model’s output. A higher sensitivity index indicates that small changes in the parameter lead to significant variations in the output, whereas a lower index suggests greater system stability. In this study, the sensitivity analysis is conducted to analyze the behaviour of $\mu '_{xy}$ (24), $\nu '_{xy}$ (25), and $\Pi _{xy}$ (27) employed in the fuzzy aggregation operators $\oplus _{1}$ (29), $\oplus _{2}$ (31), $\oplus _{3}$ (33), and $\oplus _{4}$ (34) concerning the parameter $\Upsilon$.

The sensitivity analysis is conducted in two ways: (i) The sensitivity index has been calculated for the DDD-1 image from Figure 4 for all the functions and operators. $\mu '_{xy}$ (24), $\nu '_{xy}$ (25), $\Pi _{xy}$ (27), $\oplus _{1}$ (29), $\oplus _{2}$ (31), $\oplus _{3}$ (33), and $\oplus _{4}$ (34). (ii) The average sensitivity index has been calculated for the entire BDD100k^58^ dataset for all the functions and operators. $\mu '_{xy}$ (24), $\nu '_{xy}$ (25), $\Pi _{xy}$ (27), $\oplus _{1}$ (29), $\oplus _{2}$ (31), $\oplus _{3}$ (33), and $\oplus _{4}$ (34). The sensitivity indexes are tabulated in Tables 3 and 4 as follows:

**Fig. 12:**
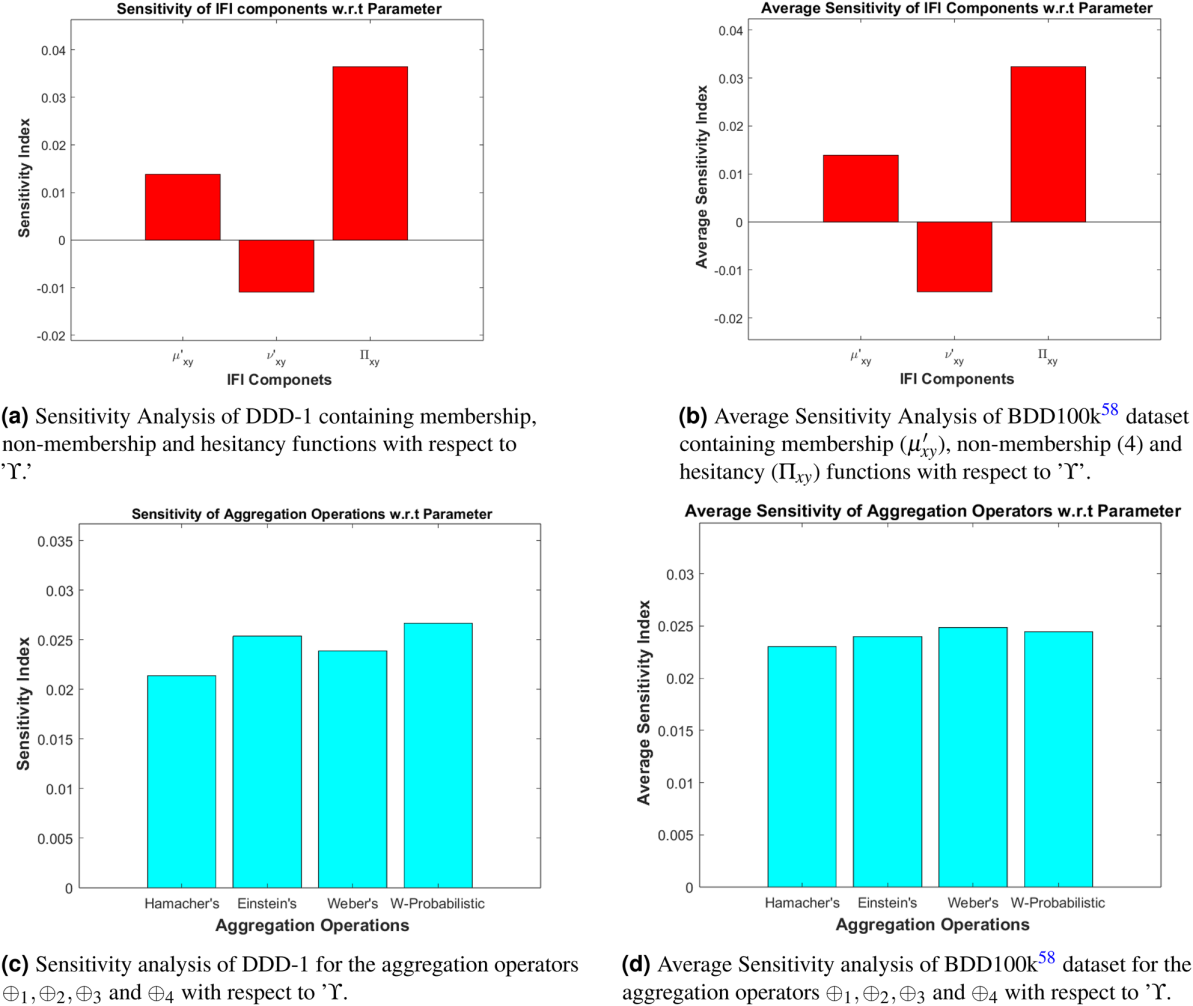
Graphical representation of the sensitivity analysis.

**Table 3:** Sensitivity analysis of $\mu '_{xy}$, $\nu '_{xy}$ and $\Pi _{xy}$ with respect to ’$\Upsilon$.

	Membership ($\mu '_{xy}$)	Non-membership ($\nu '_{xy}$)	Hesitation ($\Pi _{xy}$)
Sensitivity Index	0.0138	− 0.0110	0.0365
Average Sensitivity Index	0.0139	−0.0145	0.0323

**Table 4:** Sensitivity analysis of $\oplus _{1}, \oplus _{2}, \oplus _{3}$ and $\oplus _{4}$ with respect to ’$\Upsilon$.

	Hamacher’s $\oplus _{1}$	Einstein’s $\oplus _{2}$	Webers’s $\oplus _{3}$	W-Probablistic $\oplus _{4}$
Sensitivity Index	0.0214	0.0254	0.0239	0.0138
Average ﻿Sensitivity index	0.0230	0.0240	0.0249	0.0244

In Fig. 12, the overall sensitivity analysis is graphically presented, showcasing the responsiveness of various fuzzy components and aggregation operators to changes in the scalar parameter $\Upsilon$. The sensitivity index calculated reveals the greater robustness and stability of the proposed methodology.

### Limitations of the proposed methodology

- The experimental validation is largely limited to specific night-time driving datasets, which may not capture the full variability encountered in real-life autonomous driving conditions in which it’s generalizability remains uncertain.
- The method relies on an IFG reformed by a specific increasing function from the Sugeno class. However, the behavior and enhancement performance of the model are sensitive with respect to the IFG involved.
- Utilizing inappropriate increasing or decreasing functions may lead to inconsistent results while computing the membership and non-membership values of the intuitionistic fuzzy image (IFI), affecting the stability and adaptability of the system across different visual inputs.
- This method purely works on image enhancement perspective and does not account for multi-modal sensor inputs like LIDAR, RADAR and thermal imaging.

## Conclusion

This study demonstrates the effectiveness of intuitionistic fuzzy generating functions combined with T-Norm and T-Conorm operators for enhancing night-time images in autonomous driving systems. By leveraging the ability of intuitionistic fuzzy logic to represent uncertainty more comprehensively, the proposed approach achieves significant improvements in contrast enhancement, noise suppression and illumination correction. Experimental results on night-time driving datasets confirm that our method outperforms conventional enhancement techniques by preserving critical visual details and improving overall image clarity. These findings suggest that integrating intuitionistic fuzzy logic with T-Norm and T-Conorm operations is a significant approach for enhancing autonomous vehicle perception in low-light environments, ultimately contributing to safer and more reliable night-time driving. Further, this method can be embedded into vision modules of autonomous vehicles to enhance image clarity during night-time and low-visibility conditions thereby improving the performance of downstream tasks such as pedestrian tracking, obstacle and lane detection. Beyond ADSs, this approach can be applied to surveillance and security networks to monitor dim-light environments. Future research may focus on real-time implementation like multi-modal sensor fusion and further optimization to enhance its applicability in autonomous navigation systems via hyper-parameter tuning.

## Data Availability

The data utilized for this study are available at [https://doi.org/10.48550/arXiv.1805.04687]
